# New Insights Concerning Phytophotodermatitis Induced by Phototoxic Plants

**DOI:** 10.3390/life14081019

**Published:** 2024-08-16

**Authors:** Cristina Grosu (Dumitrescu), Alex-Robert Jîjie, Horaţiu Cristian Manea, Elena-Alina Moacă, Andrada Iftode, Daliana Minda, Raul Chioibaş, Cristina-Adriana Dehelean, Cristian Sebastian Vlad

**Affiliations:** 1 Department of Toxicology, Drug Industry, Management and Legislation, Faculty of Pharmacy, “Victor Babeș” University of Medicine and Pharmacy Timisoara, 2nd Eftimie Murgu Square, 300041 Timisoara, Romania; cristina.grosu@umft.ro (C.G.); jb.robert.alex@gmail.com (A.-R.J.); alina.moaca@umft.ro (E.-A.M.); andradaiftode@umft.ro (A.I.); cadehelean@umft.ro (C.-A.D.); 2 University Clinic Clinical Skills, Department I Nursing, Faculty of Nursing, “Victor Babes” University of Medicine and Pharmacy Timisoara, 2nd Eftimie Murgu Square, 300041 Timisoara, Romania; 3 Timisoara Municipal Emergency Clinical Hospital, 5 Take Ionescu Bv., 300062 Timisoara, Romania; 4 Research Centre for Pharmaco-Toxicological Evaluation, “Victor Babeș” University of Medicine and Pharmacy, 2nd Eftimie Murgu Square, 300041 Timisoara, Romania; 5 Department of Pharmacognosy, “Victor Babes” University of Medicine and Pharmacy Timisoara, 2nd Eftimie Murgu Square, 300041 Timisoara, Romania; daliana.minda@umft.ro; 6 Research and Processing Center for Medical and Aromatic Plants (Plant-Med), “Victor Babeş” University of Medicine and Pharmacy, 2nd Eftimie Murgu Square, 300041 Timisoara, Romania; 7 Faculty of Medicine, “Victor Babes” University of Medicine and Pharmacy Timisoara, 2nd Eftimie Murgu Square, 300041 Timisoara, Romania; chioibas.raul@umft.ro; 8 CBS Medcom Hospital, 12th Popa Sapca Street, 300047 Timisoara, Romania; 9 Department of Biochemistry and Pharmacology, Faculty of Medicine, “Victor Babes” University of Medicine and Pharmacy Timisoara, 2nd Eftimie Murgu Square, 300041 Timisoara, Romania; vlad.cristian@umft.ro

**Keywords:** phototoxicity, photosensitizers, furanocoumarins, psoralens, coumarins, ultraviolet radiation, *Heracleum mantegazzianum*, *Pastinaca sativa*, *Citrus* × *aurantifolia*

## Abstract

The present review explores the underlying mechanisms of phytophotodermatitis, a non-immunologic skin reaction triggered by certain plants followed by exposure to ultraviolet radiation emitted by sunlight. Recent research has advanced our understanding of the pathophysiology of phytophotodermatitis, highlighting the interaction between plant-derived photosensitizing compounds (e.g., furanocoumarins and psoralens) and ultraviolet light leading to skin damage (e.g., erythema, fluid blisters, edema, and hyperpigmentation), identifying these compounds as key contributors to the phototoxic reactions causing phytophotodermatitis. Progress in understanding the molecular pathways involved in the skin’s response to these compounds has opened avenues for identifying potential therapeutic targets suitable for the management and prevention of this condition. The review emphasizes the importance of identifying the most common phototoxic plant families (e.g., Apiaceae, Rutaceae, and Moraceae) and plant species (e.g., *Heracleum mantegazzianum*, *Ruta graveolens*, *Ficus carica*, and *Pastinaca sativa*), as well as the specific phytochemical compounds responsible for inducing phytophototoxicity (e.g., limes containing furocoumarin have been linked to lime-induced photodermatitis), underscoring the significance of recognizing the dangerous plant sources. Moreover, the most used approaches and tests for accurate diagnosis such as patch testing, Wood’s lamp examination, or skin biopsy are presented. Additionally, preventive measures such as adequate clothing (e.g., long-sleeved garments and gloves) and treatment strategies based on the current knowledge of phytophotodermatitis including topical and systemic therapies are discussed. Overall, the review consolidates recent findings in the field, covering a diverse array of phototoxic compounds in plants, the mechanisms by which they trigger skin reactions, and the implications for clinical management. By synthesizing these insights, we provide a comprehensive understanding of phytophotodermatitis, providing valuable information for both healthcare professionals and researchers working to address this condition.

## 1. Introduction to Phytophotodermatitis

Phytophotodermatitis is a skin condition caused by exposure to certain plants or plant-based products followed by exposure to sunlight. The combination of plant chemicals and ultraviolet (UV) light leads to a skin reaction, which can range from mild redness and irritation to severe blistering and peeling [1].

In the following chapters, we will explore the latest research and insights concerning phytophotodermatitis, including its causes, symptoms, diagnosis, and treatment options. Additionally, we will discuss the plants commonly associated with phytophotodermatitis and provide recommendations for prevention and management.

### 1.1. Brief Explanation of Phytophotodermatitis and Historical Background

Phytophotodermatitis is a skin condition induced by exposure to phototoxic plants containing photosensitizing substances such as furanocoumarins, followed by ultraviolet light exposure [2]. This non-immunologic reaction occurs due to the combination of photosensitizing agents contained in plants and ultraviolet radiation [3]. Plants contain compounds that can be toxic to the skin upon direct contact, causing phytodermatitis, or become phototoxic when exposed to ultraviolet radiation, causing phytophotodermatitis [4,5]. Psoralens and furanocoumarins are found to be the key phototoxic agents found in various plant species [6]. Certain plants like citrus fruits (*Citrus* sp.), celery (*Apium graveolens*), and wild parsnip (*Pastinaca sativa*) contain these photosensitizing compounds, among others, leading to phytophotodermatitis [7].

Historically, phytophotodermatitis has been associated with lime-induced hyperpigmentation and other plant-related cases [3]. The condition presents as a burning, painful rash with blisters on the affected skin upon exposure to UVA rays [2]. The clinical presentation also includes hyperpigmentation and blister formation, resembling other dermatological conditions like allergic contact dermatitis [8]. Additionally, the condition can mimic other dermatological skin issues, leading to potential misdiagnosis [9]. Activities like farming, bartending (handling limes), and some herbal remedies have been linked to phytophotodermatitis cases [10,11].

Phytophotodermatitis, a well-recognized condition nowadays, has historical roots dating back to ancient times, notably in ancient Egypt around 2000 BC where psoralens were used with sunlight to treat vitiligo [12]. The term “phytophotodermatitis” gained prominence in the 20th century as cases were documented in the medical literature, particularly among individuals handling plants containing phototoxic chemicals like psoralens and being exposed to ultraviolet light. The nomenclature was formally introduced by Dr. Robert Klaber in 1942 to delineate this particular phototoxic reaction elicited by plant-derived compounds upon interaction with ultraviolet radiation [12,13]. This phenomenon has captivated the interest of medical practitioners and researchers alike, prompting investigations into the pathophysiology, clinical manifestations, diagnostic criteria, and management strategies associated with this intriguing dermatosis [7]. This condition, characterized by skin inflammation after contact with plants and subsequent sunlight exposure, has been observed in various settings such as agriculture, outdoor activities, and therapeutic practices [10,11].

Throughout history, phytophotodermatitis has been associated with various plant families such as Rutaceae, Apiaceae, Moraceae, Asteraceae, and Fabaceae. Notably, plants like *Heracleum mantegazzianum* (giant hogweed), *Pastinaca sativa* (wild parsnip), and *Citrus × aurantifolia* (lime) have garnered attention for their ability to induce severe skin phototoxic reactions in individuals [14]. The understanding of phytophotodermatitis has advanced over time due to progress in chemistry and medicine, leading to the identification of furanocoumarins as the primary photosensitizers responsible for this condition. This discovery has significantly aided in unraveling the pathogenesis and clinical manifestations of phytophotodermatitis [15]. The condition’s name itself, derived from “phyto” (plant), “photo” (light), and “dermatitis” (skin inflammation), underscores its etiology [16,17]. The recognition of this condition has evolved, emphasizing the importance of identifying and avoiding contact with plants containing phototoxic substances, especially in regions where these plants are prevalent [18,19,20].

### 1.2. Epidemiology and Incidence Rate of Phytophotodermatitis

The incidence of phytophotodermatitis varies depending on factors such as geographical location, climate, and occupational plant exposure. Commonly reported cases involve reactions to plants containing furanocoumarins and psoralens, which are known to induce phototoxicity upon exposure to sunlight [7,15,21]. Children are particularly susceptible to phytophotodermatitis due to recreational exposure in nature, with psoralen-containing plants being a common cause of phototoxic dermatitis in this age group [11].

Epidemiological studies have shown that the incidence of phytophotodermatitis varies geographically, with higher rates in regions abundant in phototoxic plants due to warm and humid climates [22,23]. Phytophotodermatitis is notably prevalent among individuals engaged in activities that entail direct interaction with plants, such as gardening, farming, and landscaping, workers in plant processing industries, and individuals who come into contact with various types of plants including wild species, horticultural specimens, crops, ornamental plants, and their essential oils [10,11,24]. The risk of developing phytophotodermatitis is notably increased in these occupational groups due to the frequent and close contact they maintain with a diverse range of plants that possess phototoxic properties, thereby increasing the likelihood of adverse skin reactions upon subsequent exposure to sunlight. This occupational hazard underscores the importance of recognizing the potential risks associated with working closely with plants having phototoxic properties and implementing appropriate preventive measures to mitigate the incidence of phytophotodermatitis among at-risk populations [24,25].

Furthermore, the development of phytophotodermatitis can be influenced by factors such as homemade remedies and exposure to plant-derived photosensitizing compounds due to the increasing popularity of herbal medicine and natural remedies. Occupational and recreational activities involving plant contact have also been associated with an increased risk of phytophotodermatitis [11,15]. Healthcare providers must be aware of the different epidemiologic patterns and incidence rates of phytophotodermatitis in various regions to effectively identify and manage cases of this concerning condition due to its potential to cause serious skin damage and discomfort [4,6].

### 1.3. Etiology and Pathophysiology of Phytophotodermatitis

It is well-known that phytophotodermatitis occurs when the skin comes into contact with photosensitizing compounds found in certain plants, followed by exposure to sunlight, particularly UVA rays, triggering a phototoxic reaction on the skin. The primary culprits behind the etiology of phytophotodermatitis are natural photosensitizers, which are present in various common and wild plant species such as fig leaves (*Ficus carica*) or wild parsnip (*Pastinaca sativa*) [5,7,26,27]. These photosensitizers can sensitize the skin to ultraviolet light, leading to a range of symptoms from painful burns and rashes to blisters and edema [7,28,29].

The pathophysiology of phytophotodermatitis involves a two-step process. Initially, the skin is exposed to the photosensitizing compounds contained in the plants upon contact. These compounds penetrate the skin and remain in a latent state until activated by exposure to sunlight, specifically UVA radiation (320–380 nm). Upon UVA exposure, these compounds undergo a photochemical reaction, leading to the generation of reactive oxygen species and subsequent damage to the skin cells [11,12,21]. This process triggers an inflammatory response in the skin, resulting in the characteristic symptoms of phytophotodermatitis, including erythema, blistering, and pain [30,31].

It is essential to note that phytophotodermatitis is non-allergic contact dermatitis, meaning that the reaction is not mediated by the immune system but rather by the direct toxic effects of the photosensitizing compounds on the skin cells [18,32]. The severity of the reaction can vary depending on factors such as the concentration of the photosensitizing compounds, the duration of sunlight exposure, and individual skin sensitivity [18]. Additionally, certain plant species like giant hogweed (*Heracleum mantegazzianum*) have been reported to cause severe phytophotodermatitis, leading to full-thickness chemical burns that may require surgical intervention [31].

## 2. Overview of Phototoxic Plants

### 2.1. Common Phototoxic Plant Families and Species

The presence of phototoxic compounds in plants is a widespread phenomenon, with over 40 families, representing 32 orders and 8 subclasses of the Magnoliophyta, reported to exhibit phototoxic activity [24,33]. Coumarins and furanocoumarins are among the top chemical families with many phototoxic compounds, emphasizing their significance in plant-induced phytophotodermatitis. These compounds, along with quinones, anthraquinones, naphtodianthrones, alkaloids, saponins, and lectins, contribute to the phototoxic effects observed in various plant species [24]. 

Among the various plant families known for their phototoxic properties, the Apiaceae (also known as Umbelliferae) family stands out as one of the most well-known [12,24]. Furocoumarins, a type of psoralen, are prevalent in plants from the Apiaceae family, including popular vegetables like celery (*Apium graveolens*), wild parsnip (*Pastinaca sativa*), and carrot (*Daucus carota*), making them potent inducers of phototoxic reactions [34,35]. Additionally, the Rutaceae family, which includes citrus fruits, is another significant contributor to phototoxicity due to the presence of furocoumarins in its plant species. These families are recognized for their high content of phototoxic compounds that can cause skin reactions upon exposure to sunlight [24,36]. Moreover, furocoumarins, such as xanthotoxin (8-MOP) and bergapten, have been isolated from other plant families including Moraceae, Fabaceae, Rosaceae, and Asteraceae [37].

Furthermore, the Asteraceae family, which includes a wide range of flowering plants, has also been identified for its phototoxic potential, with compounds like polyacetylenes found in these plants, known to exhibit phototoxic properties [38,39].

The Hypericaceae family, including species like *Hypericum perforatum* (St. John’s Wort), has also been identified for its phototoxic effects due to the naphthodianthrone compounds presence, which act as photosensitizers. These compounds can induce phototoxic reactions in individuals exposed to plants from the Hypericaceae family, highlighting the diverse range of plant families possessing phototoxic properties [12,40].

Table 1 presents a comprehensive overview of the main phototoxic families and the identified phototoxic compound classes within them. The table utilizes a color-coded system to highlight the presence of metabolite classes in botanical families. Specifically, the darker green color represents the specific presence of these metabolite classes, while the lighter green color indicates the non-specific presence of the metabolites. This visual representation aids in quickly identifying which botanical families contain phototoxic compounds and the extent of their presence within these families. Table 1 serves as a valuable resource for researchers and professionals in the fields of pharmacology, botany, and dermatology, providing a structured and organized summary of phototoxic compound classes across various plant families.

The photosensitizing compounds’ presence in plants underscores the importance of understanding the distribution of phototoxic components across different plant families to identify potential sources of phototoxic reactions and implement preventive measures to mitigate the risks associated with plant exposure. By recognizing the phototoxic potential of plants from these families and understanding the mechanisms underlying their phototoxicity, measures can be taken to minimize the risk of skin damage associated with exposure to these plants.

### 2.2. Distribution and Habitat of Phototoxic Plants

Phototoxic plants, which are plants capable of causing skin reactions when exposed to sunlight, are distributed worldwide, with a significant number belonging to families like Apiaceae, Rutaceae, and Moraceae [37]. These plants are commonly found in temperate and subtropical regions, with some being present in tropical areas. The distribution of phototoxic plants is influenced by various factors such as climate, soil type, and altitude [41]. Furthermore, the habitat preferences of phototoxic plants are often influenced by factors such as light availability and herbivore pressure. Plants containing phototoxic compounds like furanocoumarins are commonly found in open habitats such as swamps, roadsides, waste places, and wet meadows where they may serve as a defense mechanism against herbivores [41,42]. The growth and distribution of these plants are shaped by interactions with herbivores, with some montane species exhibiting lower distribution boundaries due to differences in herbivore pressure [43].

Certain phototoxic plants have specific environmental requirements and are more common in particular habitats, affecting the likelihood of human exposure to phytophotodermatitis [44]. Understanding the geographical distribution of phototoxic plants is essential for identifying regions where individuals may be at a higher risk of encountering them and experiencing adverse skin reactions. Additionally, the environmental factors that support the growth of these plants play a significant role in determining their abundance and potential impact on human health [18,45]. By studying the habitats where phototoxic plants thrive, researchers can better predict areas where phytophotodermatitis cases may be more prevalent and implement preventive measures to reduce the risk of exposure [46,47]. Moreover, the metabolic potential of certain plant families, such as Amaranthaceae, Anacardiaceae, Asteraceae, Fabaceae, and Rutaceae, for producing phototoxic secondary metabolites underscores the importance of considering plant biochemistry and ecology in assessing the risk of phytophotodermatitis. The biosynthesis of primary photosensitizers like coumarins, furanocoumarins, quinones, anthraquinones, and alkaloids in these plant families highlights the diverse range of compounds that can contribute to the phototoxic effects of plants [24]. Therefore, investigating the relationship between plant distribution, habitat preferences, and the production of phototoxic compounds is essential for understanding the risk of phytophotodermatitis in different regions.

For instance, giant hogweed (*Heracleum mantegazzianum*), a member of the Apiaceae family, usually grows in moist areas along riverbanks and in forests, especially in regions with a temperate climate. The plant’s tendency to thrive in these habitats raises the likelihood of people coming into contact with it while engaging in outdoor activities such as hiking or camping in these areas [48]. Similarly, fig trees (*Ficus carica*) from the Moraceae family are commonly found in Mediterranean regions with dry and sunny climates. This distribution pattern exposes agricultural workers and gardeners in these regions to the sap of fig trees, increasing the incidence of phytophotodermatitis [49]. Citrus fruits, including limes (*Citrus aurantifolia*), lemons (*Citrus limon*), and bergamot (*Citrus bergamia*) from the Rutaceae family, are cultivated in subtropical and tropical regions. They are also widely utilized in culinary and bartending activities. Consequently, individuals involved in harvesting, handling, or processing these fruits are at heightened risk of developing phytophotodermatitis [50].

Comprehending the distribution and habitat preferences of phototoxic plants spanning various plant families and ecosystems facilitates healthcare providers in evaluating the potential occupational and recreational exposure hazards for individuals inhabiting or frequenting particular geographic regions, with appropriate warning of the danger of interaction with those plants being possible.

### 2.3. Plant Parts Involved in Phytophotodermatitis

It is essential to identify the specific plants responsible for phytophotodermatitis and understand which parts of these plants can trigger skin reactions. Different plant parts contain varying concentrations of phototoxic compounds, and contact with these parts, either directly or indirectly, can lead to the development of phytophotodermatitis [51,52].

Different plant parts can contain phototoxic compounds, potentially triggering phytophotodermatitis [35,53]. The most common plant parts implicated in causing phytophotodermatitis include leaves [54], stems [55], sap [56], and fruits [57]. These plant parts may harbor compounds like coumarins, flavonoids, meroterpenes, and furanocoumarins, which are known to induce phototoxic reactions upon exposure to sunlight [24,53,58]. Understanding the distribution of phototoxic compounds in various plant parts is essential for assessing the risk of phytophotodermatitis and implementing preventive measures to reduce exposure to these compounds [59,60]. Additionally, the presence of furocoumarins and other phototoxic compounds in the leaves, stems, sap, and fruits of plants like celery (*Apium graveolens*), rue (*Ruta graveolens*), and fig trees (*Ficus carica*) underscore the importance of recognizing potential triggers for phytophotodermatitis and taking precautions to minimize the risk of skin reactions [37,61,62].

The leaves of various plants such as *Pastinaca sativa* (wild parsnip), *Heracleum maximum* (cow parsnip), *Apium graveolens* (celery), *Ficus carica* (fig), and *Citrus bergamia* (bergamot) are known to contain furocoumarins, which can induce phytophotodermatitis upon skin contact and exposure to sunlight [5,63,64,65,66]. The sap of plants such as *Heracleum mantegazzianum* (giant hogweed), *Pastinaca sativa* (wild parsnip), and *Ficus carica* (fig) is particularly potent in causing skin reactions when combined with sunlight [67,68]. The sap induces phytophotodermatitis, with skin reactions occurring when the sap is exposed to the skin and the latter is exposed to sunlight within 24–48 h. The toxic properties of the sap can persist on exposed clothing for several hours [48]. Additionally, fruits from the Rutaceae family, including *Citrus limon* (lemon), *Citrus × aurantifolia* (lime), and *Citrus aurantium* (bitter orange), are sources of furocoumarins that can lead to phytophotodermatitis [7,69]. Moreover, the stems of certain plants, such as *Toxicodendron* sp. (poison ivy), *Ruta graveolens* (rue), and *Pastinaca sativa* (wild parsnip), can also harbor furocoumarins, making them capable of causing skin reactions when in contact with sunlight [6,70,71,72,73].

Direct contact with these plant parts containing phototoxic compounds and subsequent exposure to ultraviolet light can result in symptoms of phytophotodermatitis, including redness, blistering, and hyperpigmentation [74].

## 3. Mechanisms of Action in Phytophotodermatitis

### 3.1. Photosensitizing Compounds in Plants

Photosensitizing compounds in plants play a crucial role in the development of phytophotodermatitis. Phytophotodermatitis is primarily induced by furocoumarins or psoralen classes present in plants, which become activated upon exposure to ultraviolet radiation, especially UVA [4,21,61]. These photosensitizing compounds are known to bind covalently to DNA or generate toxic oxyradicals [75]. Phototoxins are secondary metabolites found in plants that catalyze biological actions upon light absorption [76]. The phototoxicity process involves the absorption of photons by plant chemicals, leading to the formation of highly reactive excited states that can interact with biomolecules, causing toxic effects [37]. 

Plants containing these photosensitizing compounds can cause phytophotodermatitis and even full-thickness burn-like wounds when combined with ultraviolet radiation [77]. The phototoxicity of plants is not limited to humans and animals but also serves as a defense mechanism against various plant enemies, including pathogens, nematodes, and competing plant species [78].

Furanocoumarins found in various plant families such as Rutaceae, Apiaceae, Moraceae, or Fabaceae are the main photosensitizers in plants responsible for phytophotodermatitis [12,79,80]. Additionally, sesquiterpene lactones from families like Asteraceae and Lauraceae act as photosensitizing compounds [24,81].

It should be noted that furanocoumarins, polyacetylenes, and other photoactive compounds exhibit phototoxic effects that can be harnessed for their effectiveness in cancer treatment, making them potent photosensitizers for photodynamic therapy of cancer [82]. Additionally, the photosensitizing properties of furanocoumarins such as bergapten, xanthotoxin, isopimpinellin, and imperatorin (from the 8-methoxypsoralen class) are often used in the treatment of skin conditions such as vitiligo, psoriasis, eczema, or cutaneous T-cell lymphoma [37,83,84]. Psoralen, a substance commonly recognized as a photosensitizing agent, is used in a photodynamic therapy known as PUVA (=psoralen + UVA). Psoralen has also been recommended for treating alopecia [12,37,80,85].

Photopharmacology, a field rooted in photophysics and photochemistry, is based on the principles established by the laws of photochemistry. The first law, known as the Grotthuss–Draper law, states that for a photochemical change to occur, light must be absorbed by the system [86]. This light absorption activates molecules, particularly photosensitizers, essential in the photodynamic therapy process (PDT). PDT involves using photosensitizers that, upon absorbing visible light, transition to an excited state, initiating therapeutic effects [87]. The second law of photochemistry complements the first law by stating that reactions occur upon the absorption of photons during photophysical and primary photochemical processes. This photon absorption corresponds to the absorption energy due to the wave-particle duality of light, where photon absorption equals energy absorption [88].

This energy absorption is crucial in photopharmacology, where light energy triggers biologically relevant effects through the chromophore’s activation in molecules [86].

Natural photosensitizers are integral to photopharmacology, with various classes identified, such as furanocoumarins, polyacetylenes, porphyrins with chlorines, and bacteriochlorines. These photosensitizers, characterized by their intensely colored hues, absorb light due to their chromophore components, enabling them to induce photochemical reactions upon light exposure [86,89]. The absorption of light by these natural photosensitizers aligns with the principles outlined in the laws of photochemistry, emphasizing the necessity of light absorption for subsequent photochemical transformations [86]. As regards photodynamic therapy, the interaction between non-toxic photosensitizers, light, and molecular oxygen leads to the generation of reactive oxygen species, facilitating the destruction of targeted cells and tissues. This process highlights the significance of light absorption by photosensitizers in initiating therapeutic responses, following the Grotthuss–Draper law [86,90]. Overall, the laws of photochemistry provide the foundational principles for understanding how light absorption by molecules triggers photochemical changes, a concept central to fields like photopharmacology and photodynamic therapy. Using natural photosensitizers further exemplifies the practical applications of these laws in harnessing light energy for biologically relevant outcomes [86,88,89].

While molecules that absorb ultraviolet light (λ < 400 nm) appear colorless to the human eye, all other photosensitizers are intensely colored due to the chromophore part, which is responsible for the capacity of a molecule to absorb light. Ten distinct classes of natural photosensitizers have been identified in various plants. These classes encompass a wide range of compounds with unique properties that make them suitable for applications in photodynamic therapy and other fields. Among these classes are furanocoumarins, polyacetylenes, thiophenes, curcumins, xanthenoids, alkaloids (such as quinoline-alkaloids, pterins, benzyl-isoquinolines, and beta-carbolines), anthraquinones and perylene-quinones, phenalenones, porphyrins with chlorines, and bacteriochlorines. Each class offers specific characteristics that can be harnessed for their photosensitizing properties [91,92,93].

The concentration of phototoxic metabolites varies throughout the growing season, depending on the time of year, the stage of plant development, and the presence or absence of pathogens on the plant [91]. The content in photosensitizers such as coumarins can differ up to tenfold before and after flowering, as the study by Ojala et al. demonstrated for six different Apiaceae species investigated in the study [94]. Although certain plants, such as *Thyselium palustre* (milk-parsley), exhibit higher levels of certain compounds before flowering, others, like *Angelica archangelica* (angelica), demonstrate higher levels of these compounds after flowering. Research indicates that phototoxic secondary metabolites are typically undetectable or hardly detectable in uninfected, fresh plant tissues. However, their levels rise following pathogen infection [91].

Table 2 provides a comprehensive list of phototoxic plants, their families, and the identified photosensitizers. The arrangement of plant families in alphabetical order facilitates easy reference and comparison. Within each family, the species are listed alphabetically, aiding in the systematic organization of information. This tabular format enhances data accessibility related to the botanical sources of phototoxic reactions and the specific compounds accountable for photosensitivity. Table 2 is valuable for outlining the botanical sources of phototoxic reactions and the specific compounds responsible for photosensitivity. By identifying the most common plants and families known to induce phototoxicity, as well as the associated photosensitizers, Table 2 offers important insights for healthcare professionals, researchers, and individuals aiming to identify and avoid potential triggers of phototoxic reactions.

In addition to direct exposure to phototoxic plants, the use of fragrances, perfumes, cosmetic products, and dermatological preparations containing volatile oils rich in coumarins derivatives or other photosensitizing compounds can elicit phototoxic reactions. These reactions manifest as the development of pigmented streaks along the neck, predominantly concentrated in the lower region. One of the most used and well-known volatile oils in the dermato-cosmetic industry is the volatile oil of bergamot (*Citrus bergamia*), which has high bergapten content [11,12,37].

### 3.2. The Interaction between Photosensitizing Compounds in Plants and Ultraviolet Radiation

Ultraviolet radiation can be categorized into three main types: UVA, UVB, and UVC. UVA and UVB are particularly relevant to phytophotodermatitis development. UVA penetrates the skin deeply, activating photosensitizing compounds in plants, while UVB primarily causes direct DNA damage in skin cells [98,99,100]. Ultraviolet radiation plays a crucial role in the onset of phytophotodermatitis by triggering a series of biochemical and cellular reactions. Those reactions lead to the formation of reactive oxygen species and other toxic intermediates, which in turn induce damage to the skin cells and surrounding tissues. Exposure to UV light can lead to the photolysis of compounds like ergosterol, resulting in the production of photoirradiation products that contribute to the skin reactions observed in phytophotodermatitis [37,101]. Additionally, UV radiation is known to interact with living organisms at a molecular level, causing damage to cells and posing significant health risks. The harmful effects of UV radiation are well-documented, highlighting the importance of understanding the mechanisms by which UV interacts with biological systems [102].

The interaction between UV radiation and plants leading to phytophotodermatitis is a multifaceted process involving exposure to photosensitizing substances in plants followed by sunlight exposure. Understanding this interaction is crucial for implementing preventive measures like protective clothing and sunscreen, especially for those at occupational risk. Comprehensive preventive guidelines can be formulated by grasping the interplay between UV radiation and photosensitizing compounds to manage phytophotodermatitis effectively [2,11,61].

### 3.3. Cellular and Molecular Pathways Involved in Phytophotodermatitis

The cellular and molecular pathways involved in generating phytophotodermatitis are complex and multifaceted [5,37]. Photosensitizing compounds exacerbate the phototoxic effect in contact with skin and exposed to sunlight. Upon contact with the photosensitizing compounds in the plants, especially when followed by UV radiation exposure, several complex cellular and molecular events unfold, triggering the cascade of events that result in phytophotodermatitis [2,3,70].

Phytophotodermatitis induces harmful effects through Type I or Type II phototoxic responses, resulting in DNA impairment or the generation of reactive oxygen species (ROS). Type I reactions involve the formation of mono-functional adducts and bi-functional interstrand cross-links between the furan ring on the furocoumarins and pyrimidine bases of nuclear DNA. Conversely, Type II reactions instigate oxidative harm through ROS interaction with proteins, DNA, and lipids. Both responses are incited by UVA radiation. These phototoxic processes instigate mutations, cellular membrane injury, inflammation, and cell demise [11,12,103].

**Figure 1:**
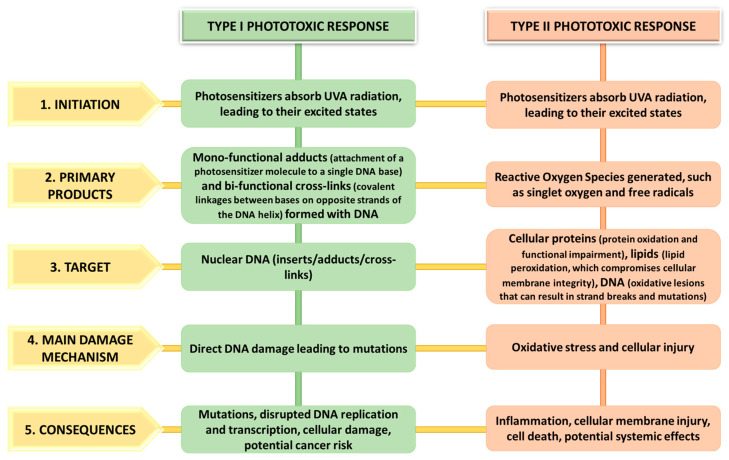
Schematic representation depicting Type I and Type II phototoxic response.

Figure 1 provides a detailed schematic representation comparing Type I and Type II phototoxic responses. The figure outlines the variances in the initiation, primary products, target, main damage mechanism, and consequences of these two types of phototoxic reactions in the context of phytophotodermatitis.

The molecular mechanisms underlying phytophotodermatitis involve cellular pathway activation, leading to skin reactions. Upon exposure to the photosensitizing substances in plants and subsequent UV light, the skin undergoes erythema, pruritus, vesiculation, and hyperpigmentation [17]. This process is independent of the immune system, highlighting the direct impact of the plant-derived compounds on skin cells. The skin reactions are the result of the complex interplay between the plant compounds, UV radiation, and the skin cells’ response mechanisms [2,12,104,105].

Reactive oxygen species (ROS) play a crucial role in skin damage induced by various environmental factors, including ultraviolet (UV) exposure. Upon exposure to UV radiation, photosensitizing compounds undergo photochemical reactions, leading to the generation of ROS within skin cells. These ROS are highly reactive and can instigate damage to lipids, proteins, and DNA, contributing to the cellular injury observed in conditions like phytophotodermatitis [11,12,103,105]. Furthermore, ROS generation is stimulated by UVB light, with enzymes like catalase directly participating in the production of oxidants in response to UVB exposure [106]. To counteract the detrimental effects of ROS, the skin has evolved antioxidant defense mechanisms, including enzymes like superoxide dismutase, glutathione peroxidase, and catalase [107]. Additionally, the transcription factor Nrf2 has been identified as a critical regulator in the skin’s response to UV radiation, establishing a glutathione-mediated cytoprotective gradient in the epidermis [108]. Studies have also explored the protective effects of various compounds against UV-induced skin damage. For instance, compounds like galangin and hesperidin have shown protective effects against oxidative stress and apoptosis induced by UV radiation [109,110]. Moreover, pre- and post-treatment with vitamin C has been demonstrated to suppress UVB-induced skin cell damage, apoptosis, DNA damage, and inflammatory responses [111].

In the pathogenesis of phytophotodermatitis, the activation of inflammatory pathways is a critical factor. This process involves the interaction between photosensitizing compounds and UV radiation, leading to the release of inflammatory mediators like cytokines and chemokines. Subsequently, this triggers an inflammatory cascade characterized by vasodilation, increased vascular permeability, and the recruitment of immune cells to the exposed site. These events culminate in the manifestation of erythema, edema, and blistering, which are typical features observed in phytophotodermatitis [53,112,113]. Photosensitization, following the absorption of photons by chromophores in the skin, is a fundamental mechanism of UV-induced oxidative stress [114]. Photosensitized reactions, which can occur through electron transfer or hydrogen abstraction (type I mechanism) and/or the production of singlet molecular oxygen (type II mechanism), contribute significantly to skin damage induced by UVA radiation [44,103,114,115]. Furthermore, the oxidation of biomolecules like proteins and nucleotides induced by photosensitizers plays a crucial role in developing skin conditions and diseases [116,117].

The direct interaction of furanocoumarins and other sensitizers with DNA leads to the formation of covalent DNA adducts, disrupting DNA replication and transcription, which can result in genetic mutations and cellular dysfunction. The impairment of DNA repair mechanisms exacerbates cellular damage, contributing to the pathophysiology of phytophotodermatitis [12,118,119]. Research has demonstrated that furanocoumarins present in various plants can induce phototoxic effects, causing skin irritation and potentially leading to conditions like phytophotodermatitis [120,121].

Understanding the cellular and molecular pathways involved in phytophotodermatitis is crucial for developing targeted interventions. By elucidating the mechanisms underlying the skin’s response to phototoxic plant exposure and UV radiation, healthcare providers can customize treatment strategies to address oxidative stress, inflammation, and DNA damage, thereby enhancing the management of phytophotodermatitis [12,122,123]. Additionally, the noise in cellular signaling networks resulting from protein–protein interactions has been shown to influence cellular signal transduction, affecting the skin’s response to phototoxic agents [124]. Moreover, the phototoxic potential of various substances, including drugs and plant-derived compounds, underscores the importance of understanding and mitigating phototoxic reactions for effective dermatological management [12,125,126].

## 4. Clinical Presentation and Diagnosis of Phytophotodermatitis

The clinical presentation of phytophotodermatitis can vary, with some cases manifesting as asymptomatic hyperpigmentation without preceding inflammation, adding to the complexity of diagnosis and recognition [21,127,128].

**Figure 2:**
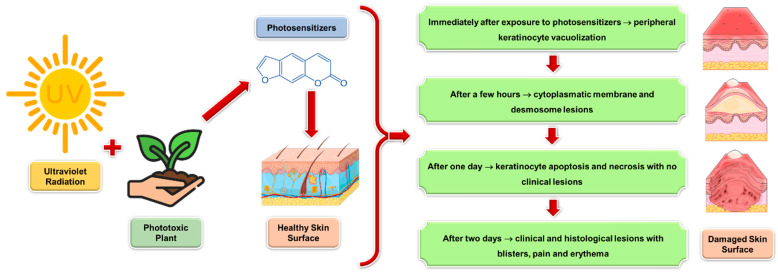
Schematic representation of the developmental stages of phytophotodermatitis. The figures in the diagram were created using the Flaticon platform and Servier Medical Art (licensed under Creative Commons Attribution 3.0 Unported License).

Figure 2 provides a schematic representation of the developmental stages of phytophotodermatitis. The figure likely illustrates the progression of symptoms and skin changes, which occur following exposure to plant photosensitizers. It may include stages such as initial erythema, followed by blister formation, and possibly hyperpigmentation or skin discoloration as the reaction resolves. Understanding the developmental stages of phytophotodermatitis is crucial for accurate diagnosis and management of this condition, which often requires avoiding further exposure to phototoxic plant substances and appropriate skin care to promote healing and prevent complications.

### 4.1. Cutaneous Manifestations

Phytophotodermatitis requires a thorough clinical and etiological examination to ensure accurate diagnosis and treatment [129,130]. The condition typically presents with symptoms appearing a few hours or days after contact with photosensitizing plants and subsequent sun exposure [11,53]. The primary causative agents in phytophotodermatitis are psoralens found in various plants, including citrus fruits and fig trees, which, when activated by ultraviolet light, lead to skin reactions [20,54].

The clinical presentation of phytophotodermatitis can vary, with acute phototoxic reactions characterized by erythema, edema, and hyperpigmentation [4,12]. Additionally, asymptomatic hyperpigmentation without preceding inflammation has been reported as a clinical feature of citrus fruits-induced phytophotodermatitis [4]. Moreover, phytophotodermatitis can mimic other conditions, such as non-accidental injury or self-harm, highlighting the importance of accurate diagnosis [9]. Plants containing phototoxic agents can induce a variety of skin pathologies, including contact, allergic, and phytophotodermatitis, as well as skin pigmentation defects due to altered melanogenesis [131].

The initial sign of cutaneous manifestations of phytophotodermatitis often involves erythema, indicating an inflammatory response induced by photosensitizing compounds, which can progress to edema and, in severe cases, blister and bullae formation due to the combined effects of these compounds and UV radiation. These vesicular eruptions are a result of the inflammatory cascade and the damage to the skin’s structural integrity caused by the combined effects of the phototoxic compounds and UV radiation [11,21,129]. 

The cutaneous manifestations of phytophotodermatitis appear as linear streaks or irregular patterns of erythema and blistering on sun-exposed areas of the body that have been in contact with the plant, reflecting the imprint of phototoxic plant parts (such as leaves or stems). Some individuals can also present splashed marks if the phototoxic reaction is triggered by the juice of citrus fruits. These typically manifest within 24–48 h post-exposure, although delayed onset up to 2–3 days is possible with phototoxic plant contact. Predilection sites for lesion development include the hands, forearms, arms, and legs, with potential involvement of the chest, abdomen, lips, and hips [1,11,27,61,129,132].

Phytophotodermatitis is clinically characterized by an erythema, or redness, accompanied by a burning sensation and fluid blisters, as well as local itching and edema. It is often described as erythematous, edematous, and well-demarcated, with crepuscular plaques that can develop bullous vesicles. The lesions are usually painful or burning rather than itchy, which may be an important feature in distinguishing it from allergic contact dermatitis. The rash can persist for months or even years and may result in post-inflammatory hyperpigmentation. The sensitivity to ultraviolet light may persist. The pathogenic mechanism of the condition involves the absorption of radiant energy by photosensitive substances, which then transfer the energy to other molecules, causing inflammatory skin phenomena. The rash has a unique appearance, with intersecting lines at different angles, and may include erythema-to-vesicular lesions, demarcated lines, papule-like lesions, and irregularly shaped plaques [11,12,129,132].

Experimental investigations on animal models have revealed that 24 h post-exposure, histological alterations manifest as vacuolization and keratinocyte necrosis, progressing to vesicle formation by the 48-h mark. These changes are accompanied by observable erythema and vesiculation. Initial damage occurs at the cellular membrane and desmosomes. Epithelial degeneration is observable through light microscopy, while early changes, such as keratinocyte vacuolization and membrane lesions, are detectable via transmission electron microscopy. Following experimental phytophotodermatitis induction, initial evaluations at the cellular and clinical levels indicate normal epidermal conditions, with necrosis becoming apparent after 24 h and evolving into epidermal vesicles within 48 h. The delay between the injurious stimulus and cellular manifestations aligns with the principles of cell death and apoptosis, akin to reactions seen in drug-induced injuries. Additionally, in vitro, cell culture models exposed to psoralen and type A ultraviolet radiation also exhibit induced cellular apoptosis [17].

Post-inflammatory hyperpigmentation arises from multiple mechanisms, such as the leakage of pigment due to epidermal cell death, an increase in the number of functional melanocytes and melanosomes, an increase in the branching of melanocytes, and an upsurge in tyrosine’s activity. This condition is comparable to that observed in PUVA therapy, and the associated hyperpigmentation may persist for 1 to 2 years [27,37].

By promptly identifying the clinical features of phytophotodermatitis, healthcare providers can initiate appropriate management and preventive measures to alleviate symptoms and prevent further exacerbation of the condition. It is important to consider the possibility of phytophotodermatitis in patients presenting with these skin reactions, especially if there is a history of plant contact followed by sun exposure. 

### 4.2. Differential Diagnosis

Diagnosis of phytophotodermatitis is generally made on a clinical basis, with history taking being extremely important in identifying exposures to phototoxic plants. Biopsies or photopatch tests may be performed to confirm phototoxicity, but these are not obligatory for diagnosis and only provide additional information [12].

The differential diagnosis of phytophotodermatitis involves distinguishing it from other skin conditions with similar presentations [8,9,12,133]. Differential diagnosis is often difficult because phytocompounds in plants can also cause irritant contact dermatitis or allergic contact dermatitis, not necessarily phytophotodermatitis [12,133]. Irritant contact dermatitis results from direct chemical or physical damage to the skin and may manifest as erythema, edema, and blistering similar to phytophotodermatitis. However, it can be distinguished from phytophotodermatitis by the absence of plant exposure history and the lack of a characteristic linear pattern [29,134]. Allergic contact dermatitis is an immune-mediated response that can occur within 24 to 48 h after exposure to various plants and environmental allergens, unlike phytophotodermatitis, which relies on UV radiation [29,135]. This type of dermatitis typically results from repeated exposure to chemical allergens, leading to a delayed hypersensitivity reaction mediated by T-cells [136]. The inflammatory response in allergic contact dermatitis involves a complex interplay of immune cells, cytokines, and chemokines [137]. Additionally, the skin’s innate immune system plays a crucial role in the development of allergic contact dermatitis, with sensitizing chemicals interacting with both irritant and antigenic properties to induce the condition [138].

Other diagnoses that can present with skin lesions resembling those of phytophotodermatitis should also be considered for the correct diagnosis of the condition, such as photosensitivity disorders (pellagra, solar urticaria, and porphyria), infections (tinea and herpes simplex virus), drug-induced allergic reactions, erythema multiforme, or vesiculobullous disorders [12]. One of the key differential diagnoses is melasma, a common skin condition characterized by hyperpigmented patches on the face. While plant exposure and UV light do not induce melasma, this shares similarities with phytophotodermatitis in terms of skin discoloration, highlighting the importance of accurate diagnosis [133].

In clinical practice, distinguishing phytophotodermatitis from other dermatological conditions, including allergic contact dermatitis, thermal burns, and infectious skin diseases, is essential to providing targeted treatment and preventing misdiagnosis [12,139]. 

Overall, the differential diagnosis of phytophotodermatitis involves recognizing its distinct clinical features and distinguishing them from other dermatological conditions with similar cutaneous manifestations induced by plants, chemicals, or environmental factors. By meticulously evaluating patient history, conducting skin patch testing, and considering the temporal sequence of plant exposure and subsequent UV radiation, healthcare providers can accurately diagnose and manage phytophotodermatitis, thus preventing misdiagnosis and inappropriate treatment interventions, ensuring optimal patient outcomes [11,12].

### 4.3. Diagnostic Approaches and Tests

The diagnosis of phytophotodermatitis can be complex due to its diverse clinical presentations, which may manifest as burning sensations, painful rashes, blisters, and hyperpigmentation [2]. Healthcare providers can accurately diagnose phytophotodermatitis by evaluating comprehensively the patient’s clinical history of exposure to phototoxic plants [140,141]. However, in certain cases where the diagnosis is unclear or confirmation is needed, healthcare providers may consider employing diagnostic tests and approaches to support the clinical impression. A thorough physical examination focusing on the distribution of skin lesions and the presence of characteristic patterns like streaks or patches can also aid in diagnosis [2,140].

Laboratory tests are generally not necessary for diagnosing phytophotodermatitis, as the condition is primarily diagnosed based on clinical history and physical examination findings [142]. However, in cases where the diagnosis is uncertain or other potential causes of similar skin manifestations are ruled out, healthcare providers may opt for patch testing with plant extracts or suspected photosensitizing agents. Patch testing involves applying small amounts of the suspected plant extract or compound to the skin under occlusion to assess for delayed hypersensitivity reactions, which can help confirm the diagnosis of phytophotodermatitis [19,143].

In addition to history taking, physical examination, and patch testing, dermatologists may utilize Wood’s lamp examination as a diagnostic tool for phytophotodermatitis. Wood’s lamp emits ultraviolet light that can highlight areas of hyperpigmentation or fluorescence in the affected skin, aiding in the visualization of subtle skin changes that may not be visible under normal lighting conditions [2,96]. This technique can be particularly valuable in identifying persistent areas of hyperpigmentation after the acute phase of the reaction has subsided, providing further evidence of a phototoxic origin [2,140].

In some cases, a skin biopsy may be performed for diagnosing conditions like phytophotodermatitis, as it allows the examination of histopathological changes in the affected skin. Typical findings include epidermal spongiosis, vesicle formation, and dermal inflammatory infiltrates, which are characteristic of this condition [144,145,146]. The histopathological examination aids in the accurate diagnosis and identification of etiological agents and guides appropriate management decisions [147]. 

It is important to note that the diagnosis of phytophotodermatitis may be challenging due to its resemblance to other dermatological conditions. Therefore, a multidisciplinary approach involving dermatologists, allergists, and photobiologists may be beneficial in formulating a comprehensive diagnostic strategy and ensuring the accurate identification of phytophotodermatitis.

## 5. Treatment and Management Strategies for Phytophotodermatitis

In severe cases of phytophotodermatitis, multidisciplinary collaboration between medical professionals from different specialties (dermatology, surgery, emergency medicine, and burn units) is needed to choose an appropriate treatment regimen. Severe cases may require hospitalization, rehydration of intravenous fluid, systemic steroids, and transfer to a specialized burn center. In the burn unit, general sedation and pain relief may be necessary to facilitate wound assessment. If the lesion thickens, it might call for skin grafts and potential amputation. It is crucial to continuously monitor for secondary infections, which might require systemic antibiotics. As lesions formed after vesicle rupture can be considered chemical burns, they should be assessed on an ongoing basis [11,12]. 

The initial steps in starting therapy involve removing and steering clear of any substances that can cause sensitivity to light and/or ultraviolet rays. Once exposure is identified, the affected area should be cleaned with soap and water. Since epidermal necrosis often leads to pain, certain patients might need pain relief using non-steroidal anti-inflammatory drugs (NSAIDs) or opioids. If there are signs of inflammation such as redness or swelling, applying topical steroids may help manage the symptoms [11,12,25,129]. It is advisable to use sunscreens to prevent further damage and protect the skin from heightened sensitivity following exposure to harmful substances. Activities that expose the affected area to moisture (e.g., swimming) can worsen the lesions and should be avoided [12,71].

If large, ruptured blisters have occurred, debridement is necessary, but the following aspects should be taken into account: the location of the lesion and risk of infection, the potential for healing, the need to assess wound depth, patient comfort, clinician experience, moisture balance, mechanical pressure, the application of dressings, and medication. Small blisters can be drained using a sterile needle. Superficial lesions can be cleaned with chlorhexidine, and topical antibiotics, such as silver sulfadiazine, may be applied. Wound dressing is necessary to reduce pain, aid in re-epithelization and healing, and protect against infection and UV radiation. Pressure bandages can be used over wound dressings if there is edema. A moisturizing cream or ointment can also be applied to help regenerate the wound [11,12].

Residual hyperpigmentation is common and can be treated with topical steroids, hydroquinone, azelaic acid, and tretinoin to help reduce unwanted discoloration or by various procedures including chemical peels and laser treatments, although these do carry some risk of worsening hyperpigmentation or causing visible hypopigmentation. It is essential to avoid sun exposure and further protect the affected area. Prompt removal of the photosensitizing agent, local and general therapy, and photoprotective measures ensure a prompt therapeutic response in most cases [11,12,71,129,148].

### 5.1. Topical Therapies

The management of phytophotodermatitis depends on the severity of the skin injury, with mild cases often requiring only symptomatic treatment [149]. In the management of phytophotodermatitis, topical therapies play a crucial role. Various treatments can be employed to alleviate symptoms and promote healing. Topical corticosteroids are commonly used to reduce the inflammation and itching associated with phytophotodermatitis [11,150,151,152]. Additionally, emollients can help maintain skin hydration and promote skin barrier repair [5,150]. For severe cases, systemic treatments like immunosuppressive agents may be necessary [151]. It is essential to tailor the treatment approach based on the severity of the condition and individual patient characteristics.

Topical corticosteroids are commonly used to alleviate the inflammatory response and reduce symptoms associated with phytophotodermatitis. These medications work by suppressing the immune response and mitigating the release of inflammatory mediators in the skin. Healthcare providers may prescribe low- to mid-potency corticosteroid creams or ointments for mild to moderate cases, while severe cases may necessitate higher-potency formulations under close medical supervision [5,6,11,25,153]. Topical corticosteroids are effective in treating phytophotodermatitis, especially in the acute phase, by helping to reduce inflammation and symptoms [153].

Topical antihistamines are beneficial in managing pruritus and discomfort in phytophotodermatitis cases. They can alleviate itching and relieve skin irritation, especially in areas with vesicular eruptions and edema. Both first- and second-generation antihistamines are commonly used to suppress pruritus and prevent exacerbation due to scratching, making them valuable in dermatological conditions like phytophotodermatitis [11,154]. Additionally, the synergistic effect of H1-antihistamines with topical corticosteroids has been highlighted, emphasizing their role in treating pruritus in atopic dermatitis. The use of antihistamines, either alone or in combination with other medications, is essential in managing pruritus associated with various skin conditions, providing relief, and improving the quality of life for affected individuals [155].

Applying cool compresses to areas affected by phytophotodermatitis can help alleviate symptoms by reducing inflammation and soothing the skin. This practice is recommended by healthcare providers to minimize the inflammatory response and aid in the healing of skin lesions [11,149]. 

Emollients play a crucial role in maintaining skin hydration and aiding in barrier repair post-phytophotodermatitis. The regular application of emollients can help prevent excessive dryness, reduce skin cracking, and support healing [156]. Emollients that contain urea, ceramide nanoparticles, and lactate have been shown to enhance skin hydration and improve barrier function. Additionally, emollients with glycerol can enhance skin hydration by increasing their moisturizing capacity [157]. Topically applied lipids in emollients can aid in skin barrier repair [158]. Furthermore, the combination of glycerol and petrolatum in emollients has been found to enhance skin barrier function and hydration [159].

Calamine lotion, a mixture of zinc oxide and ferric oxide, is commonly recommended for its soothing properties in treating conditions like phytophotodermatitis. It acts by alleviating itching and forming a protective layer on the skin. Studies have shown its efficacy in managing various dermatological issues [160]. The lotion’s application can aid in symptom relief and enhance comfort during the acute phase of phytophotodermatitis. Additionally, calamine lotion has been suggested for managing chronic urticaria and other skin conditions due to its calming effects. Its use, supported by experts, is prevalent in Indian clinical settings for addressing pruritus and chronic urticaria [161]. Furthermore, the lotion has been found to reduce skin irritation [162].

The use of topical calcineurin inhibitors has shown efficacy in managing phytophotodermatitis. These agents help in modulating the immune response in the skin and can be beneficial in reducing inflammation and promoting skin healing [163]. 

In cases where there is hyperpigmentation without preceding inflammation, specialized treatments focusing on addressing pigmentation changes may be required. Overall, a combination of topical therapies, systemic treatments, and patient education on plant exposure prevention and sun protection is essential in effectively managing phytophotodermatitis [11,61]. 

Healthcare providers should closely monitor patients’ response to topical therapies, assessing for secondary infections and adjusting treatment regimens as needed [164]. Follow-up appointments are crucial for identifying complications early and providing appropriate interventions. Topical treatments can effectively manage mild cases of various skin conditions, offering advantages such as fewer contraindications [165]. While systemic antibiotics are vital for infected wounds, using topical antiseptics may prevent biofilm formation [166]. Careful consideration is necessary when deciding on systemic or topical antibiotics for wound management [167].

### 5.2. Systemic Treatments

A systemic treatment modality that has been considered is the use of corticosteroids to reduce inflammation and alleviate symptoms associated with phytophotodermatitis [61]. Corticosteroids can help in managing the inflammatory response triggered by the phototoxic agents present in plants, thereby reducing redness, swelling, and discomfort [168]. Additionally, systemic corticosteroids may aid in preventing the progression of the reaction and promoting faster resolution of skin manifestations [5]. Systemic corticosteroids, such as prednisone, are used in severe cases of phytophotodermatitis to reduce inflammation and immune responses, particularly in scenarios involving extensive blistering. However, it is crucial to closely monitor patients due to the potential systemic side effects associated with corticosteroid use. The monitoring and management of complications linked to corticosteroid therapy, especially in critical conditions, are fundamental aspects of patient care [70,169]. 

Another systemic treatment option that has been proposed is the use of systemic antihistamines to manage itching and discomfort associated with phytophotodermatitis [11]. Antihistamines can help in controlling the histamine release triggered by the inflammatory process, thereby reducing itching and promoting patient comfort [170]. By alleviating itching, antihistamines can also prevent excessive scratching, which may further irritate the skin and exacerbate the condition [171].

In severe cases of phytophotodermatitis, systemic treatments such as immunomodulators may be considered to modulate the immune response and promote healing [172]. Immunomodulators can help regulate the immune system’s reaction to phototoxic agents, potentially reducing the severity of the skin manifestations and accelerating the recovery process. By targeting the immune response, these treatments aim to address the underlying mechanisms contributing to the development of phytophotodermatitis [173].

Patient education is crucial in empowering individuals to manage conditions like phytophotodermatitis effectively. Regular follow-up visits are essential for treatment continuation, identifying misdiagnoses, managing complications, and ensuring patient compliance.

### 5.3. Supportive Care and Symptomatic Relief

Phytophotodermatitis induced by phototoxic plants is a condition that can lead to significant discomfort due to skin damage. In the management of phytophotodermatitis, supportive care and symptomatic relief play a crucial role in alleviating the associated symptoms [11,12]. Supportive care in this context involves a multidisciplinary approach aimed at addressing the distressing symptoms caused by the condition. This approach includes effective communication, shared decision-making, and advance care planning. Additionally, it focuses on providing relief from pain and integrating psychological and spiritual aspects of care to help patients cope with the illness and its effects [174].

Symptomatic relief for phytophotodermatitis is essential in improving the quality of life of affected individuals [174]. Treatments such as topical corticosteroids, cool compresses, and pain management can help alleviate the discomfort and inflammation associated with the condition. Furthermore, the use of emollients and moisturizers can aid in skin healing and reduce the risk of secondary infections. It is important to note that the management of phytophotodermatitis may also involve addressing complications such as blistering and hyperpigmentation, for which specific treatments may be required based on the severity of the symptoms [11,175].

By adopting a multidisciplinary approach that focuses on addressing distressing symptoms, providing relief from pain, and integrating psychological support, healthcare providers can effectively support individuals affected by this condition and improve their quality of life. The comprehensive management of phytophotodermatitis encompasses a range of treatment modalities, preventive strategies, and supportive care measures aimed at addressing both the acute manifestations and long-term impact of the condition. By integrating various interventions and providing ongoing support, healthcare providers can guide patients toward effective symptom management, skin protection, and overall well-being in the context of phytophotodermatitis.

### 5.4. Protective Measures and Precautions

Protective measures and precautions against phytophotodermatitis are crucial in preventing and managing skin reactions caused by phototoxic plants. Understanding the importance of personal protection is essential in minimizing the risk of phytophotodermatitis. Workers exposed to phototoxic plants should adhere to strict protective measures to avoid skin damage. This includes wearing appropriate personal protective equipment such as gloves that cover the forearms, protective masks with goggles to prevent eye irritation, long-sleeved waterproof garments, and masks to reduce inhalation of plant sap or aerosols [10,11,25,175].

In the context of healthcare settings, the use of personal protective equipment has been highlighted as a key protective measure. Studies have shown that workers who consistently use personal protective equipment, such as masks and gloves, demonstrate a lower risk of phytophotodermatitis [25,175]. Proper hygiene practices, including regular handwashing, have also been emphasized as effective protective measures, including against plant-induced dermatitis [176,177].

Moreover, the application of broad-spectrum sunscreen has been recommended as a preventive measure against UV-induced skin damage. Sunscreen helps to mitigate the harmful effects of UV radiation, which can exacerbate skin reactions caused by phototoxic plants. By incorporating sunscreen into daily skincare routines, individuals can provide an additional layer of protection against the damaging effects of UV exposure [178]. Additionally, wearing protective clothing like long-sleeved shirts, wide-brimmed hats, and sunglasses can offer extra defense against UV radiation, reducing the risk of phytophotodermatitis. Sunscreen, when correctly applied, can prevent acute sunburn and should be combined with other protective measures such as clothing, hats, and seeking shade [175,176,178,179]. 

Educating individuals on identifying phototoxic plants and promoting awareness of species causing phytophotodermatitis is crucial for prevention. Familiarity with plant features like leaf patterns and growth habits can help avoid contact with phototoxic plants. Strategies like using designated pathways and avoiding foliage contact during outdoor activities can reduce the risk of phototoxic reactions [12].

Implementing specific occupational precautions in agricultural, horticultural, or landscaping settings is crucial to reduce the risk of phytophotodermatitis. Utilizing protective gear like gloves, long pants, and closed-toe shoes can act as a barrier against direct skin contact with phototoxic plants. Employers and health professionals play an essential role in promoting workplace safety and educating employees on recognizing and preventing plant-induced phototoxic reactions. Safety coaching can be effective in enhancing safety knowledge and hazard recognition in the agricultural sector [11,25,177,180]. 

Healthcare providers, public health organizations, and community stakeholders working together to promote preventive measures can substantially help decrease plant-induced phototoxic reactions in the population.

## 6. Conclusions and Future Research Directions

Phytophotodermatitis induced by phototoxic plants represents a fascinating intersection between botany, dermatology, and environmental health, with wider implications for both clinical practice and public awareness. Continuing to investigate the complexity of plant-induced phototoxicity through interdisciplinary research may pave the way for innovative approaches to the prevention, diagnosis, and treatment of this dermatological condition. The future of phytophotodermatitis research holds great promise for advancing the understanding of plant–skin interactions, improving patient care, and ultimately alleviating the burden of this not fully understood but clinically significant phenomenon. The exploration of novel therapeutic approaches and the ongoing advancements in the understanding and management of phytophotodermatitis represent a fundamental change in the dermatological care landscape.

The present review article discusses the complex mechanisms underlying phytophotodermatitis induced by phototoxic plants, emphasizing the pathophysiology, clinical manifestations, diagnosis, and management of this dermatological condition. Through an in-depth review of the literature, it is evident that the interactions between plant-derived psoralens or furocoumarins and ultraviolet radiation play an essential role in the occurrence of phytophotodermatitis. The characteristic erythema, edema, and vesicles that occur in affected individuals are the result of a complex cascade of events involving DNA damage, inflammatory responses, and oxidative stress. Through disclosure of these molecular cascades, researchers can identify new therapeutic targets for managing and ameliorating the symptoms of phytophotodermatitis, thereby improving clinical outcomes and patient care. The recent findings in the field consolidate the existing knowledge base on phytophotodermatitis, providing a comprehensive overview of the diverse phototoxic compounds found in plants, their mechanisms of action, and the implications for clinical management. 

In the future, new research directions in the field of phytophotodermatitis should focus on improving the understanding and management of this condition. First, the elucidation of the precise molecular pathways involved in the phototoxic reaction induced by specific plant compounds will be essential for the development of targeted therapies and preventive strategies. Advanced molecular techniques such as gene expression profiling and proteomics can provide valuable insights into the complex interplay between plant-derived photosensitizers, skin cells, and immune responses. Furthermore, exploring the role of genetic predisposition in determining individual susceptibility to phytophotodermatitis could lead to the identification of new personalized medical approaches in the future. Furthermore, the development of new diagnostic tools for the rapid and accurate identification of phytophotodermatitis is essential to facilitate early intervention and prevent long-term sequelae. Incorporating imaging modalities such as confocal reflection microscopy or optical coherence tomography into clinical practice can help visualize the depth and extent of skin damage caused by phototoxic plants. In addition, the establishment of standardized guidelines for the management of phytophotodermatitis, including recommendations for decontamination, topical therapies, and aftercare, is imperative to ensure optimal patient outcomes and minimize the complications of this condition. Clinicians should maintain a high index of suspicion for phytophotodermatitis, particularly in cases that present with a linear or striated pattern of skin lesions after plant contact and sun exposure.

Overall, this review research serves as a fundamental contribution to the dermatology field by synthesizing the latest research on phytophotodermatitis, providing a holistic perspective on its etiology, pathophysiology, and management. Nevertheless, the role of botanic gardens in educating people about phototoxic plants and promoting safe interactions with nature should not be underestimated. These institutions can serve as valuable resources for disseminating plant toxicity information by collaborating with institutions that research plant–skin interactions, thus highlighting the indigenous flora with phototoxic properties in a controlled environment. Collaborations between academia, health institutions, and botanic gardens could lead to improved collective knowledge of phytophotodermatitis and its implications for human health and well-being.

## Figures and Tables

**Table 1:** The main phototoxic families and the identified phototoxic compound classes.

	Plant Phototoxic Compound Class									
Plant Family	Alkaloids	Catechols	Coumarins, Furanocoumarins	Lectins	Polyphenols	Porphyrins and Derivatives	Quinones, Anthraquinones, Naphtodianthrones	Saponins	Tannins	Thiophenes
Amaranthaceae										
Anacardiaceae										
Annonaceae										
Apiaceae										
Asteraceae										
Fabaceae										
Hypericaceae										
Lamiaceae										
Poaceae										
Rubiaceae										
Rutaceae										

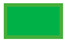
 the presence of the specific metabolite classes, 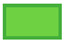 the presence of the non-specific metabolites.

**Table 2:** The main phototoxic families and plant species with identified photosensitizers.

Plant Family	Plant Species	Plant Photosensitizers	Ref.
Amaranthaceae	*Alternanthera* *philoxeroides* (Alligator Weed)	anthraquinone—rubiadin, rubiadin-1-methyl-ether, 2-hydroxy3-methyl-anthraquinone, psoralens—angelicin, bergapten	[95]
	*Froelichia humboldtiana* (Mexican Fireweed)	psoralens—bergapten	[95]
	*Spinacia oleracea* (Spinach)	chlorophyll derivatives, chlorophyllin	[95]
Anacardiaceae	*Toxicodendron* sp. (Poison Ivies)	urushiol	[25]
Annonaceae	*Annona purpurea*	berberine	[95]
Apiaceae (Umbelliferae)	*Ammi majus* (Queen Anne’s Lace)	8-methoxypsoralen: xanthotoxin, bergapten, ammirin, imperatorin, alloimperatorin, marmesin, marmesinin, oxypipeucedanin	[12,95,96]
	*Ammi visnaga* (Khella)	8-methoxypsoralen: xanthotoxin, 8-hydroxy-bergapten, imperatorin, marmesin	[95]
	*Anethum graveolens* (Dill)	furanocoumarins, 5-methoxypsoralen	[12,37]
	*Angelica archangelica* (Angelica)	furanocoumarins, angelicin	[12]
	*Angelica sylvestris* (Wild Angelica)	furanocoumarins, angelicin	[12]
	*Anthriscus sylvestris* (Cow Parsley)	furanocoumarins	[2,12]
	*Apium graveolens* (Celery)	furanocoumarins, bergapten	[2,12,37,96]
	*Chaerophyllum* *macropodum*	8-methoxypsoralen	[12]
	*Cymopterus watsonii*	xanthotoxin, bergapten, imperatorin	[95]
	*Daucus carota* (Carrot)	furanocoumarins, psoralen	[2,12,37]
	*Ferula orientalis*	ferujol	[12]
	*Foeniculum vulgare* (Fennel)	furanocoumarins, psoralen	[2,12,37]
	*Heracleum lanatum* (Cow Parsnip)	furanocoumarins	[12]
	*Heracleum* *mantegazzianum* (Giant Hogweed)	furanocoumarins	[2,12,37,96]
	*Heracleum persicum* (Persian Hogweed)	furanocoumarins	[12]
	*Heracleum sosnowskyi* (Sosnowskyi’s Hogweed)	furanocoumarins	[12]
	*Heracleum sphondylium* (Cow Parsley)	furanocoumarins	[12]
	*Notobubon galbanum* (Hog’s Fennel)	galbanin	[12]
	*Pastinaca sativa* (Parsnip)	xanthotoxin, bergapten, imperatorin, isopimpinellin	[2,12,37,95,96]
	*Petroselinum crispum* (Parsley)	furanocoumarins, myristicin	[37]
	*Peucedanum paniculatum*	peucenin	[12]
	*Pimpinella anisum* (Anise)	furanocoumarins, anethole	[12]
Araceae	*Aglaonema simplex* (Chinese Evergreen)	1-hydroxypurpurin-7-lactone ethyl-methyl-diester	[95]
Asphodelaceae	*Aloe vera* (Aloe)	aloe-emodin, aloin	[62,95]
Asteraceae (Compositae)	*Chrysanthemum indicum* (Chrysanthemum)	sesquiterpene lactones	[25]
	*Echinops exaltatus* (Globe Thistle)	sesquiterpene lactones (echinopsin, cnicin, and cynaropicrin)	[12]
	*Echinops latifolius*	thiophenes, sesquiterpene lactones (echinopsin, cnicin, and cynaropicrin)	[12]
	*Tagetes patula* (French Marigold)	thiarubrine, thiophenes	[12]
Berberidaceae	*Mahonia aquifolium* (Oregon Grape)	berberine	[12,37]
Brassicaceae (Cruciferae)	*Brassica napus, Brassica rapa* (Canola)	methyl isothiocyanate	[95]
Chenopodiaceae	*Chenopodium album* (Lamb’s Quarters)	unknown	[12]
Fabaceae (Papilionaceae, Leguminosae)	*Biserrula pelecinus* (Snail Clover)	unknown	[95]
	*Bituminaria bituminosa* (Bituminous or Rock Clover)	psoralen, angelicin	[95]
	*Cicer arietinum* (Chickpea)	coumarins	[12,37]
	*Cullen cinereum*	psoralens—bergapten	[95]
	*Glycine max* (Soybean)	furanocoumarins	[12,37]
	*Lotus corniculatus* (Bird’s-foot Trefoil)	furanocoumarins	[95]
	*Medicago nigra* (Black Medick)	furanocoumarins	[95]
	*Medicago sativa* (Alfalfa)	furanocoumarins	[95]
	*Myroxylon balsamum* (Balsam of Peru)	psoralens—bergapten	[12]
	*Pisum sativum* (Pea)	coumarins	[12,37]
	*Psoralea corylifolia* (Babchi)	psoralens—angelicin, bergapten	[12]
	*Trifolium pratense* (Red Clover)	coumarins—melilotoside	[95]
	*Trifolium repens* (White Clover)	furanocoumarins	[95]
Geraniaceae	*Erodium moschatum, Erodium cicutarium*	8-methoxypsoralen, 5-methoxypsoralen, imperatorin	[95]
Hypericaceae	*Hypericum perforatum* (St. John’s Wort), *Hypericum tetrapterum* (St. Peter’s Wort), *Hypericum perfoliatum*	hypericin, psoralens	[12,95,96]
Lamiaceae	*Scutellaria barbata* (Skullcap)	pheophorbide A	[95]
Malvaceae	*Malachra fasciata* (Yellow False Mallow)	bergapten, xanthotoxin, isopimpinellin, imperatorin	[95]
Moraceae	*Ficus carica* (Common Fig)	furanocoumarins: psoralens—bergapten, methoxsalen	[2,12,37,96]
	*Ficus pumila* (Creeping Fig)	furanocoumarins: psoralens—methoxsalen	[12]
Myrtaceae	*Syzygium aromaticum* (Clove)	eugenol, furanocoumarins	[91]
Papaveraceae	*Chelidonium majus* (Greater Celandine)	protoberberine, furocoumarins, psoralen, bergapten, isopimpinellin, chelerythrine	[95]
Plantanginaceae	*Plantago lanceolata* (Ribwort Plantain)	unknown	[12,97]
Poaceae (Gramineae)	*Echinochloa frumentacea, E. esculenta, E. crus-gall* (Barnyard Grass)	psoralens	[95]
Polygonaceae	*Fagopyrum esculentum* (Buckwheat)*, Fagopyrum tataricum*	perilenquinone—fagopyrin	[12]
Ranunculaceae	*Helleborus niger* (White Hellebore or Christmas Rose)	protoanemonin, hellebrin, berberine	[12,95]
	*Hydrastis canadensis* (Goldenseal)	hydrastine, berberine	[12,37]
	*Ranunculus* sp.	ranunculin	[12]
Rubiaceae	*Coffea arabica* (Coffee)	unknown	[12,37]
	*Gardenia* sp.	geniposide, crocetin	[12,37]
	*Heterophyllaea pustulata, Heterophyllaea lycioides*	psoralens—angelicin	[12,95]
Rutaceae	*Citrus aurantium* (Bitter Orange)	furanocoumarins	[12]
	*Citrus bergamia* (Bergamot Orange)	furanocoumarins	[2,12,37,96]
	*Citrus latifolia* (Persian Lime)	furanocoumarins	[12]
	*Citrus limetta* (Sweet Lemon)	furanocoumarins	[12]
	*Citrus limon* (Lemon)	furanocoumarins	[2,12,37,96]
	*Citrus maxima* (Pomelo)	furanocoumarins	[12]
	*Citrus medica* (Citron)	furanocoumarins	[12]
	*Citrus paradisi* (Grapefruit)	furanocoumarins	[12]
	*Citrus sinensis* (Sweet Orange)	furanocoumarins	[12]
	*Citrus × aurantifolia* (Lime)	furanocoumarins	[2,12,37,96]
	*Dictamnus albus* (Burning Bush)	linear furanocoumarins, psoralen	[12,96]
	*Pelea anisata* (Mokihana)	furanocoumarins	[12]
	*Ptelea crenulata* (California Hoptree)*, Ptelea* *trifoliata* (Water Ash)	psoralen, bergapten	[12]
	*Rhadinothamnus anceps* (Blister Bush)	furanocoumarins	[12]
	*Ruta graveolens* (Common Rue)	psoralen, bergapten, isopimpinellin, isoimperatorin, chalepensin, marmesin, isorutarin	[12,95,96]
	*Thamnosma texana* (Texas Rue)	psoralen, xanthotoxin, bergapten, isopimpinellin	[95]
Solanaceae	*Capsicum annuum* (Chili Pepper)	unknown	[12,37]
Zingiberaceae	*Curcuma longa* (Turmeric)	curcumin, turmerone	[95]

## Data Availability

The data supporting the findings of the present review are available within the article.
