# Correction: Grosu et al. New Insights Concerning Phytophotodermatitis Induced by Phototoxic Plants. *Life* 2024, *14*, 1019

**DOI:** 10.3390/life15030337

**Published:** 2025-02-21

**Authors:** Cristina Grosu (Dumitrescu), Alex-Robert Jîjie, Horaţiu Cristian Manea, Elena-Alina Moacă, Andrada Iftode, Daliana Minda, Raul Chioibaş, Cristina-Adriana Dehelean, Cristian Sebastian Vlad

**Affiliations:** 1 Department of Toxicology, Drug Industry, Management and Legislation, Faculty of Pharmacy, “Victor Babeș” University of Medicine and Pharmacy Timisoara, 2nd Eftimie Murgu Square, 300041 Timisoara, Romania; cristina.grosu@umft.ro (C.G.); jb.robert.alex@gmail.com (A.-R.J.); alina.moaca@umft.ro (E.-A.M.); andradaiftode@umft.ro (A.I.); cadehelean@umft.ro (C.-A.D.); 2 University Clinic Clinical Skills, Department I Nursing, Faculty of Nursing, “Victor Babes” University of Medicine and Pharmacy Timisoara, 2nd Eftimie Murgu Square, 300041 Timisoara, Romania; 3 Timisoara Municipal Emergency Clinical Hospital, 5 Take Ionescu Bv., 300062 Timisoara, Romania; 4 Research Centre for Pharmaco-Toxicological Evaluation, “Victor Babeș” University of Medicine and Pharmacy, 2nd Eftimie Murgu Square, 300041 Timisoara, Romania; 5 Department of Pharmacognosy, “Victor Babes” University of Medicine and Pharmacy Timisoara, 2nd Eftimie Murgu Square, 300041 Timisoara, Romania; daliana.minda@umft.ro; 6 Research and Processing Center for Medical and Aromatic Plants (Plant-Med), “Victor Babeş” University of Medicine and Pharmacy, 2nd Eftimie Murgu Square, 300041 Timisoara, Romania; 7 Faculty of Medicine, “Victor Babes” University of Medicine and Pharmacy Timisoara, 2nd Eftimie Murgu Square, 300041 Timisoara, Romania; chioibas.raul@umft.ro; 8 CBS Medcom Hospital, 12th Popa Sapca Street, 300047 Timisoara, Romania; 9 Department of Biochemistry and Pharmacology, Faculty of Medicine, “Victor Babes” University of Medicine and Pharmacy Timisoara, 2nd Eftimie Murgu Square, 300041 Timisoara, Romania; vlad.cristian@umft.ro

In the published publication [1], there was an error regarding the affiliation for Horaţiu Cristian Manea. In replacement of affiliation 2, the updated affiliation should be as follows: University Clinic Clinical Skills, Department I Nursing, Faculty of Nursing, “Victor Babes” University of Medicine and Pharmacy Timisoara, 2nd Eftimie Murgu Square, 300041 Timisoara, Romania. In replacement of personal email address: office@medcom.ro, the updated email address should be as follows: chioibas.raul@umft.ro, which is the institutional address.

The authors state that the scientific conclusions are unaffected. This correction was approved by the Academic Editor. The original publication has also been updated.
